# Abnormal Brain MRI Findings in Anti-*N*-Methyl-D-Aspartate Receptor Encephalitis and Correlation With Outcomes

**DOI:** 10.3389/fneur.2022.834929

**Published:** 2022-03-14

**Authors:** Chunyan Lei, Xiaolong Chang, Haijiang Li, Lianmei Zhong

**Affiliations:** Department of Neurology, First Affiliated Hospital of Kunming Medical University, Kunming, China

**Keywords:** anti-NMDAR encephalitis, brain MRI, clinical characteristic, clinical outcome, cognitive function

## Abstract

**Purpose:**

The reported prevalence of abnormal findings by brain MRI varies from 11 to 83% among patients with anti-*N*-methyl-D-aspartate receptor (NMDAR) encephalitis. Here, we investigated the prevalence of abnormal MRI findings in Chinese patients and explored whether such findings are correlated with clinical outcomes.

**Methods:**

This retrospective study analyzed a consecutive series of 52 patients with anti-NMDAR encephalitis admitted to our hospital. The patients were assigned to the “MRI-normal” or the “MRI-abnormal” group based on brain MRI after admission. The groups were compared in terms of clinicodemographic characteristics and scores on the Mini-Mental State Examination (MMSE) and modified Rankin Scale (mRS) 3 and 12 months after admission.

**Results:**

Thirty-seven (71.15%) of the patients showed abnormalities on brain MRI; these patients were more likely to be men and showed abnormalities on electroencephalography. Patients who showed normal or abnormal MRI findings did not differ significantly in terms of clinical symptoms, rates of mortality or relapse, or mRS scores after 3 and 12 months. However, patients with abnormal MRI showed significantly lower MMSE scores than those with normal MRI after 3 and 12 months.

**Conclusions:**

We found high prevalence of abnormal MRI findings in our sample of Chinese patients with anti-NMDAR encephalitis. We also found that the abnormal findings were associated with cognitive decline but not necessarily with mortality or functional outcomes in the short or long term.

## Introduction

In anti-*N*-methyl-d-aspartate receptor (NMDAR) encephalitis, autoimmune antibodies bind and inactivate the NMDAR, which plays pivotal roles in synaptic transmission and plasticity (1, 2). Anti-NMDAR encephalitis can manifest as various syndromes, including seizures, autonomic instability, neuropsychiatric symptoms, memory disorder, dyskinesia, movement disorder, disturbed consciousness, and speech disorders. Although anti-NMDAR encephalitis can be cured, immunotherapy has not been standardized (1–4).

The disease is typically diagnosed based on detection of anti-NMDAR antibodies in cerebrospinal fluid and/or serum, together with clinical characteristic, laboratory findings, brain MRI, and electroencephalography (3–5). Studies have reported quite different prevalence of abnormal brain MRI findings among patients with anti-NMDAR encephalitis ranging from 11 to 83% (6, 7). The abnormalities typically occur in the cerebral or cerebellar cortex, hippocampus, and/or insular and frontobasal regions (6, 7).

Therefore, we conducted a retrospective study on patients in our hospital in order to measure the prevalence of abnormal brain MRI findings, and to explore whether these findings are correlated with symptoms or outcomes. We also compared clinical and radiological characteristics among patients who experienced different outcomes.

## Methods

### Patients

Medical records were retrospectively analyzed for patients with anti-NMDAR encephalitis admitted to the First Affiliated Hospital of Kunming Medical University between January 1, 2016 and October 31, 2018 who underwent contrast-enhanced brain MRI. The patients were diagnosed based on clinical manifestations and the presence of anti-NMDAR IgG antibody in the cerebrospinal fluid (4, 5). All specimens (cerebrospinal fluid and serum) were assessed for anti-NMDAR IgG antibodies by indirect immunofluorescence using EU 90 cells transfected with the NMDAR1 subunit (NR1) of the NMDAR complex and immobilized on BIOCHIPs (Euroimmun AG, Lübeck, Germany) as previously reported (7). Patients diagnosed with infectious encephalitis or encephalitis due to other known causes were excluded in the study.

The study was approved by the Ethics Committee of the First Affiliated Hospital of Kunming Medical University, which waived the requirement for informed consent because at the time of admission, the patients or their relatives provided written consent for patients' anonymized medical data to be analyzed and published for research purposes.

### Clinical Evaluations

An experienced neurologist recorded patients' clinical symptoms according to eight previously reported manifestations (4, 5): abnormal behavior, speech disorder, movement disorder, seizures, sleep disturbance, memory disorder, altered consciousness, and central hypoventilation. Consciousness was assessed using the Glasgow Coma Scale (GCS). Data were collected on history of autoimmune diseases, length of hospital stay, duration of immunotherapy since disease onset, admission to intensive care unit, electroencephalography, and treatments. Data were also collected on results of laboratory tests for white blood cell count and protein concentration in cerebrospinal fluid, as well as presence of anti-NMDAR antibody in the cerebrospinal fluid or serum. The patients were examined for the presence of tumors by ultrasonography and computed tomography of the pelvis and thoraco-abdominal region.

### Brain MRI

**Figure 1:**
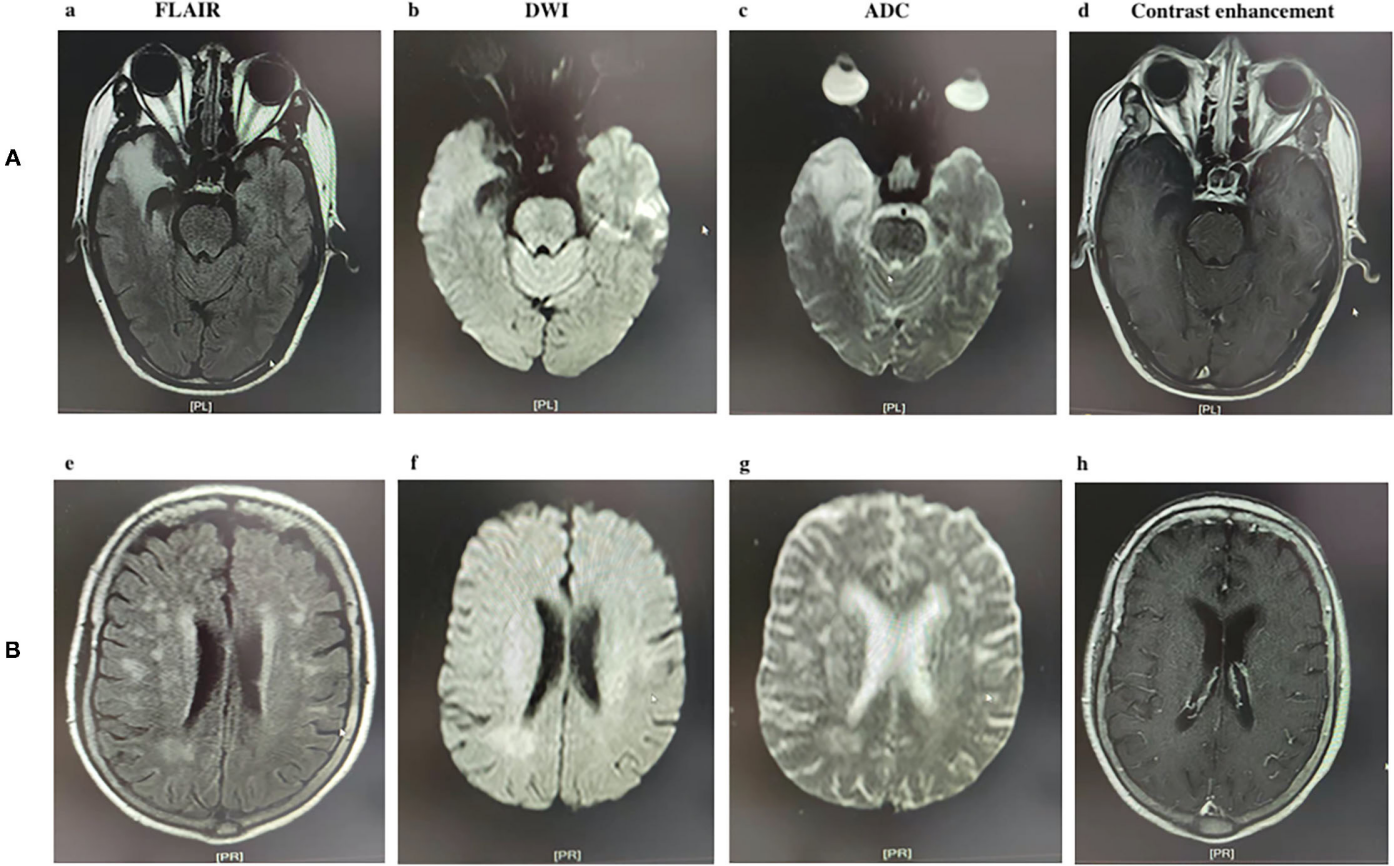
Two types of MRI image appearance in patients with anti-N-methyl-D-aspartate (NMDA) receptor encephalitis. **(A)** A 45-year-old male patient with lesions located in the right temporal lobe; (a) axial fluid-attenuated inversion recovery (FLAIR) image showing high signal; (b) Diffusion-weighted imaging showing slightly high signal; (c) Apparent diffusion coefficient image showing high signal, (d) no contrast enhancement is evident in this axial T1 sequence. **(B)** A 22-year-old female patient with lesions located in the white matter; (e) axial FLAIR image showing high signal; (f) DWI image showing slightly high signal; (g) ADC image showing slightly high signal, (h) no contrast enhancement is evident in this axial T1 sequence.

Brain MRI was performed using a 3.0-T Trio system (Siemens, Erlangen, Germany) and T2-weighted, T1-weighted, and fluid-attenuated inversion recovery (FLAIR) sequences. Working independently, two radiologists who were blinded to clinical data retrospectively analyzed images in order to determine whether findings were normal or abnormal. Abnormal findings were defined as T2/FLAIR signal hyperintensity in the cerebellar cortex, basal ganglia, thalamus, subcortical areas, and/or cerebral cortex, or leptomeningeal contrast enhancement (Figure 1) (6). A third neurologist was consulted in the event of disagreement.

### Outcomes

Three and 12 months after admission, the patients were evaluated by the Mini-Mental State Examination (MMSE) and modified Rankin Scale (mRS) on an outpatient basis. Poor outcome was defined as an mRS score of 3–6. Mortality was recorded by contacting family members.

### Statistical Analysis

Statistical analyses were performed using SPSS 21.0 (Chicago, IL, United States). Categorical data were reported as counts (percentages), while continuous data were reported as mean ± SD or median (interquartile range, IQR). Intergroup differences in continuous variables were assessed for significance by Student's *t-*test or Mann–Whitney *U-*test. Differences in categorical variables were assessed by Pearson's chi-squared or Fisher's exact test. Differences associated with *P* < 0.05 were considered statistically significant.

## Results

### Clinicodemographic Characteristics of the Patients

During the recruitment period, 57 patients with anti-NMDAR encephalitis were admitted to our hospital, but five were lost to follow-up, so the final analysis included 52 patients (Table 1). There were 35 (67.31%) men, and the median age across all the patients was 40 (24–44) years. Electroencephalography findings were abnormal in 22 (42.31%) of the patients, while brain MRI was abnormal in 37 (71.15%). Most patients (35, 66.04%) had received a combination of intra-venous methylprednisolone (1g/day for 5 days) and intravenous immunoglobulin (0.4 g/kg per day for 5 days), while eight (15.38) patients had received intravenous methylprednisolone and nine (17.31) patients had received intravenous immunoglobulin. Twenty (38.46%) of the patients were treated in the intensive care unit, and 11 (21.15%) had central hypoventilation. Five (9.62%) of the patients had a history of autoimmune diseases, such as rheumatoid arthritis (*n* = 3), Sjogren's syndrome (*n* = 1), and systemic lupus erythematosus (*n* = 1).

**Table 1:** Comparison of basic characteristic between the MRI-abnormal and MRI-normal groups.

**Characteristic**	**All (***N*** = 52)**	**MRI-abnormal (***N*** = 37)**	**MRI-normal (***N*** = 15)**	***P*** **value**
Age, years, (mean ± SD)	37.15 ± 12.97	39.00 ± 13.91	32.60 ± 9.09	0.058
Male	35 (67.31)	22 (59.46)	13 (86.67)	0.046
GCS score				0.011
13–15	28 (53.85)	24 (64.86)	4 (26.67)	
5–12	22 (42.31)	11 (29.73)	11 (73.33)	
3–4	2 (3.85)	2 (5.41)	0 (0.00)	
Autoimmune diseases history, (%)	5 (9.62)	4 (10.81)	1 (6.67)	0.100
Abnormal EEG	22 (42.31)	22 (59.46)	0 (0.00)	<0.001
CSF detection				
White cell count per mm^3^, (mean ± SD)	29.48 ± 50.50	24.33 ± 7.37	31.57 ± 59.80	0.474
Protein concentration, mg/dL, (mean ± SD)	0.57 ± 0.58	0.48 ± 0.62	0.61± 0.57	0.462
Positive antibody in CSF, (%)	52 (100.00)	37(100.00)	15(100.00)	NA
Positive antibody in serum, (%)	13 (25.00)	8 (21.62)	5 (33.33)	0.377
ICU admission	20 (38.46)	13 (35.14)	7 (46.67)	0.720
Central hypoventilation	11 (21.15)	7 (18.92)	4 (26.67)	0.749
Days of immunotherapy from disease onset, (mean ± SD)	25.56 ± 18.99	23.16 ± 16.76	27.19 ± 21.12	0.415
First-line therapy, (%)				
Steroids alone	8 (15.38)	2 (5.41)	6 (40.00)	0.005
Intravenous immunoglobulin alone	9 (17.31)	8 (21.62)	1 (6.67)	0.257
Combination	35(66.04)	27(72.97)	8(53.33)	0.171
Second-line therapy, (%)				
Rituximab	2 (3.85)	2 (5.41)	0 (0.00)	0.100
Cyclophosphamide	6 (11.54)	4 (10.81)	2 (13.33)	0.873

*GCS score, Glasgow coma scale; anti-NMDAR, anti-N-methyl-D-aspartate receptor; EEG, electroencephalography; CSF, cerebrospinal fluid; ICU, intensive care unit. IQR, interquartile range*.

Three-quarters (39) of the patients reported prodromal symptoms several days before the onset of the disease, which included fever (*n* = 24), headache (*n* = 24), and dizziness (*n* = 7) (Table 2). In all the 52 patients, the most common clinical manifestations were abnormal behavior (29, 55.77%), followed by seizures (25, 48.08%). Just over half of the patients showed altered consciousness, most often manifesting as confusion. A small proportion of the patients showed disorders affecting speech, movement, memory, and sleep.

**Table 2:** Comparison of clinical profile and outcomes between the MRI-abnormal and MRI-normal groups.

**Characteristic**	**All (***N*** = 52)**	**MRI-positive (***N*** = 37)**	**MRI-negative (***N*** = 15)**	***P*** **value**
Prodrome symptoms, (%)				
Fever	24 (46.15)	21 (56.76)	3 (20.00)	0.013
Headache	24 (46.15)	17 (45.95)	7 (47.67)	0.962
Dizziness	8 (15.38)	6 (16.22)	2 (13.33)	1.000
Clinical symptoms, (%)				
Abnormal behavior	29 (55.77)	18 (48.65)	11(73.33)	0.104
Movement disorder	2 (3.85)	2 (5.41)	0 (0.00)	0.100
Speech disorder	7 (13.46)	4 (10.81)	3 (20.00)	0.166
Seizures	25 (48.08)	20 (54.05)	5 (33.33)	0.175
Sleep disturbance	4 (7.69)	4 (10.81)	0 (0.00)	0.311
Memory disorder	9 (17.31)	8 (21.62)	1 (6.67)	0.257
Altered consciousness	28 (53.85)	21 (55.76)	7 (46.67)	0.509
Central hypoventilation	15 (28.85)	7 (18.92)	8 (53.33)	0.013
Based mRS, (mean ± SD)	3.23 ± 1.45	3.08 ± 1.44	3.60 ± 1.45	0.429
Clinical outcome at 3-month				
mRS, (mean ± SD)	1.52 ± 2.20	1.35 ± 2.19	1.93 ± 2.25	0.403
				0.474
mRS 0-2	35 (67.31)	26 (70.27)	9 (60.00)	
mRS3-6	17 (32.68)	11 (29.73)	6 (40.00)	
Death	5 (9.62)	3 (8.11)	2 (13.33)	0.619
Relapse	6 (11.54)	6 (16.22)	0 (0.00)	0.165
MMSE, (median, IQR)	25.00 (18.00–26.00)	21.00 (16.00–23.00)	26.00 (20.00–27.00)	0.008
Clinical outcome at 12-month				
mRS, (mean ± SD)	1.25 ± 2.01	1.11 ± 1.98	1.60 ± 2.10	0.246
				0.696
mRS 0-2	40 (76.92)	29 (78.38)	11 (73.33)	
mRS3-6	12 (23.08)	8 (21.62)	4 (26.67)	
Death	5 (9.62)	3 (8.11)	2 (13.33)	0.619
Relapse	8 (15.38)	7 (18.92)	1 (6.67)	0.412
MMSE, (median, IQR)	25 (18–27)	22 (17–24)	26 (21.5–27)	0.007

### Outcomes in the Entire Sample

In all the 52 patients, 35 (67.31%) showed good outcomes after 3 months, and this rate increased to 76.92% after 12 months. In 3 months, five (9.62%) of the patients had died and six (11.54%) had experienced relapse (Table 2). In 12 months, the number of mortalities did not increase, but relapse rate increased to 15.38%.

### Comparison of Patients With Normal and Abnormal MRI

Among the 37 patients with abnormal brain MRI, T2/FLAIR abnormal hyperintensity was observed in the cerebral cortex (*n* = 25), white matter (*n* = 4), thalamus (*n* = 2), and leptomeninges (*n* = 6). Comparison of these 37 patients with abnormal MRI to those with normal MRI showed that those with abnormal findings had worse GCS score and were more likely to be men, had abnormal electroencephalography findings, and had a history of fever (Table 1). The proportion of patients receiving steroids alone was lower in the MRI-abnormal group. The two groups did not differ significantly in the other baseline variables analyzed.

**Figure 2:**
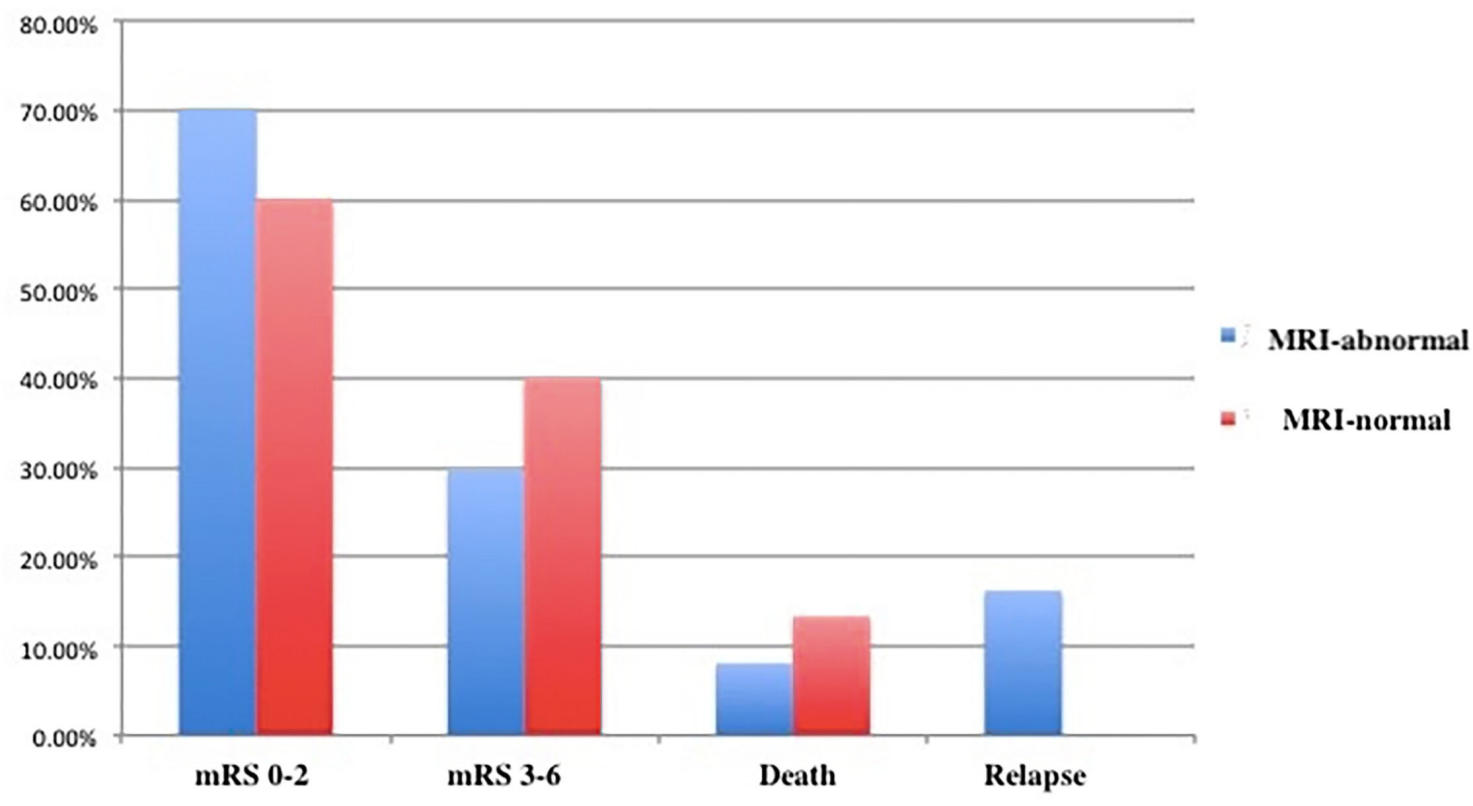
Clinical outcomes of patients with anti-NMDAR encephalitis after 3 months.

**Figure 3:**
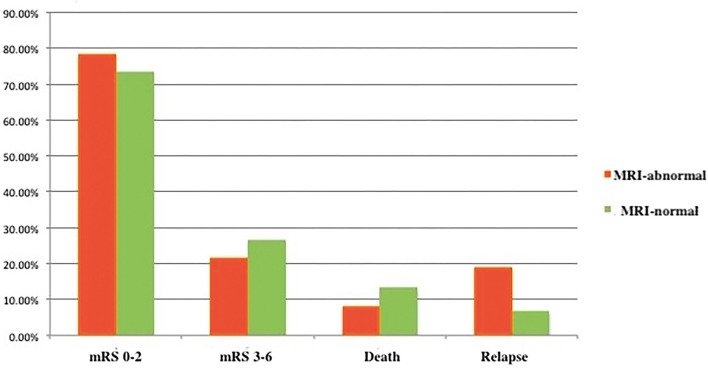
Clinical outcomes of patients with anti-NMDAR encephalitis after 12 months.

The MRI-normal and -abnormal groups did not differ significantly in mRS score or rates of mortality or relapse after 3months (Figure 2 and Table 2) or 12 months (Figure 3). However, the MMSE score at both time points was significantly lower in the MRI-abnormal group.

### Comparison of Patients Who Experienced Good or Poor Outcomes

Comparison of patients who showed good or poor outcomes after 12 months indicated that those who had poor outcomes were more likely to be men and to experience central hypoventilation (Table 3). Otherwise, the two groups did not differ significantly in the other variables examined.

**Table 3:** Comparison of clinical characteristics between the poor clinical outcome and good clinical outcome groups after 12 months.

	**Poor clinical outcome (***n*** = 12)**	**Good clinical outcome (***n*** = 40)**	***P*** **value**
Age, years, (mean ± SD)	37.67 ± 12.42	37.00 ± 13.26	0.878
Male	4 (33.33)	31 (77.50)	0.004
GCS score			0.512
13–15	6 (50.00)	22 (55.00)	
5–12	6 (50.00)	16 (40.00)	
3–4	0 (0.00)	2 (5.00)	
Abnormal electroencephalography	4 (33.33)	18 (45.00)	0.473
CSF detection			
White cell count per mm^3^, (mean ± SD)	19.42 ± 16.06	32.50 ± 56.76	0.449
Protein concentration, mg/dL, (mean ± SD)	0.68 ± 0.67	0.53 ± 0.55	0.184
Positive antibody in CSF (%)	12(100.00)	40 (100.00)	NA
Positive antibody in serum, (%)	4(33.33)	9(22.50)	0.447
ICU admission	5 (41.67)	15(37.50)	0.646
Central hypoventilation	6 (50.00)	5 (12.50)	0.065
Days of immunotherapy from disease onset, mean (mean ± SD)	23.92 ± 10.14	26.05 ± 21.01	0.736
First-line therapy, (%)			
Steroids alone	1 (8.33)	7 (17.50)	0.886
Intravenous immunoglobulin alone	1 (8.33)	8 (20.00)	0.663
Combination	10 (83.33)	25 (62.50)	0.177
Second-line therapy, (%)			
Rituximab	0 (0.00)	2 (5.00)	1.000
Cyclophosphamide	1 (8.33)	5 (12.50)	0.651
Prodrome symptoms, (%)			
Fever	3 (25.00)	21 (52.50)	0.113
Headache	6 (50.00)	18 (45.00)	1.000
Dizziness	3 (25.00)	5 (12.50)	0.366
Clinical symptoms, (%)			
Abnormal behavior	5 (41.67)	24 (60.00)	0.262
Movement disorder	0 (0.00)	2 (5.00)	1.000
Speech disorder	2 (16.67)	5 (12.50)	0.656
Seizures	6 (50.00)	19 (47.50)	0.879
Sleep disturbance	0 (0.00)	4 (50.00)	0.562
Memory disorder	1 (8.33)	8 (20.00)	0.666
Altered consciousness	8 (66.67)	20 (50.00)	0.439
Central hypoventilation	6 (50.00)	9 (22.50)	0.065
MRI findings, (%)			0.064
Normal	4 (33.33)	11 (27.50)	
Lesions in cerebral cortex	3 (25.00)	22 (55.00)	
Lesions in white matter	3 (25.00)	1 (2.50)	
Lesions in thalamus	0 (0.00)	2 (5.00)	
Lesions in Leptomeningea	2 (16.67)	4 (10.00)	

### Comparison of Patients Whose mRS Scores Changed or Not After First-Line Treatment

After 3 months of follow-up, 28 (70%) of 40 patients showed an mRS score different from their score at baseline. Patients whose mRS score did not change were more likely to be men and to experience central hypoventilation (Table 4). However, the two groups did not differ significantly in the other variables examined.

**Table 4:** Comparison of patients with no alteration in modified Rankin Scale (mRS) scores or alteration in mRS scores after first-line treatment.

	**No alteration in mRS scores** **(***n*** = 12)**	**Alteration in mRS scores** **(***n*** = 28)**	***P*** **value**
Age, years, (mean ± SD)	39.33 ± 13.50	34.51 ± 11.45	0.121
Male	4 (33.33)	25 (89.29)	0.011
GCS score			0.316
13–15	6 (50.00)	20 (71.42)	
5–12	6 (50.00)	10 (35.71)	
3–4	0 (0.00)	2 (7.14)	
Abnormal electroencephalography	4 (33.33)	16 (57.14)	0.757
CSF detection			
White cell count per mm^3^, (mean ± SD)	34.91 ± 74.01	25.38 ± 46.67	0.612
Protein concentration, mg/dL, (mean ± SD)	0.69 ± 0.66	0.57 ± 0.61	0.590
Positive antibody in CSF (%)	12 (100.00)	28 (100.00)	NA
Positive antibody in serum, (%)	4 (33.33)	7 (25.00)	0.434
ICU admission	4 (33.33)	11 (37.50)	1.000
Central hypoventilation	6 (50.00)	5 (17.86)	0.048
Days of immunotherapy from disease onset, mean (mean ± SD)	22.12 ± 9.15	27.83 ± 17.53	0.451
Prodrome symptoms, (%)			
Fever	5 (41.67)	17 (60.71)	0.498
Headache	7 (58.33)	15 (53.57)	0.498
Dizziness	2 (16.67)	4 (14.29)	0.658
Clinical symptoms, (%)			
Abnormal behavior	4 (33.33)	19 (67.86)	0.124
Movement disorder	0 (0.00)	2 (7.14)	1.000
Speech disorder	2 (16.67)	5 (17.86)	1.000
Seizures	6 (50.00)	12 (42.86)	0.214
Sleep disturbance	0 (0.00)	4 (14.29)	0.562
Memory disorder	1 (8.33)	8 (28.57)	1.000
Altered consciousness	5 (41.67)	18 (64.29)	0.504
Central hypoventilation	6 (50.00)	8 (28.57)	0.045
MRI findings, (%)			0.139
Normal	3 (25.00)	8 (28.57)	
Lesions in cerebral cortex	5 (41.67)	15 (53.57)	
Lesions in white matter	2 (16.67)	0 (0.00)	
Lesions in thalamus	0 (0.00)	2 (7.14)	
Lesions in Leptomeningea	2 (16.67)	3 (10.71)	

## Discussion

More than half our patients with anti-NMDAR encephalitis showed abnormalities on brain MRI, similar to the prevalence reported in studies on Caucasian and other Chinese patients (8, 9). However, we found evidence that such abnormalities do not seem to correlate with functional outcome or risk of mortality or relapse. On the other hand, they may be correlated with cognitive decline, as measured by the MMSE.

The literature fails to give a clear answer to the question of whether abnormal MRI findings are correlated with prognosis of patients with anti-NMDAR encephalitis. Studies on Caucasian patients have suggested that normal MRI findings are correlated with risk of seizure remission (10), and that abnormal findings are correlated with risk of poor outcome (11, 12).On the other hand, studies on Asian patients have failed to detect significant associations between MRI findings and clinical outcome (13–15). This discrepancy may reflect differences in MRI interpretation or ethnicity. Nearly 77% of the patients in our sample had a good clinical outcome, similar to the 78% of Asian patients with anti-NMDAR antibodies in another study who experienced mild sequelae or recovered (16). Anti-NMDAR encephalitis has been linked to teratoma (4), but tumors were not detected in any of our patients, or in Chinese patients in a similar study (9). The association between teratoma and the disease may depend on ethnicity, age, and sex.

We found in our sample that, regardless of whether brain MRI was normal or abnormal, patients presented similar clinical manifestations, most often abnormal behavior, followed by altered consciousness and seizures. This profile of manifestations is consistent with studies on Caucasian and Asian patients (4, 8, 12). Our results suggest that anti-NMDAR encephalitis should be suspected in any patient presenting an appropriate combination of neurological and psychiatric symptoms regardless of brain MRI findings. Such patients should then be tested for presence of anti-NMDAR antibodies. This approach, for example, may reduce the risk of misdiagnosis among patients with brain lesions in unconventional locations outside the limbic system (16).

Our study has some limitations. First, our sample size was small and had a heterogeneous treatment history, reflecting the lack of consensus on type and timing of treatment, or the duration of first- and second-line treatments. Second, we did not evaluate patients who underwent plasma exchange, which was not available in our hospital when our sample was treated.

Despite these limitations, our data suggest that normal MRI findings should not exclude a diagnosis of anti-NMDAR encephalitis, and that patients suspected of having the disease should undergo antibody testing. Our findings and their generalizability should be verified and extended in further studies.

## Data Availability Statement

The raw data supporting the conclusions of this article will be made available by the authors, without undue reservation.

## Ethics Statement

The studies involving human participants were reviewed and approved by First Affiliated Hospital of Kunming Medical University. The patients/participants provided their written informed consent to participate in this study.

## Author Contributions

CL designed the subject. HL and XC collected and extracted datad the article. LZ revised the important intelligent content. CL approved the final version of the manuscript. All authors contributed to the article and approved the submitted version.

## Funding

This study was supported by the Yunnan young and middle-aged academic and technical Training Project for High-Level Talents (202105AC160065) and Yunnan Training Project for High-Level Talents (RLQB20200003) and Basic project of Yunnan Province (202101AT070142).

## Conflict of Interest

The authors declare that the research was conducted in the absence of any commercial or financial relationships that could be construed as a potential conflict of interest.

## Publisher's Note

All claims expressed in this article are solely those of the authors and do not necessarily represent those of their affiliated organizations, or those of the publisher, the editors and the reviewers. Any product that may be evaluated in this article, or claim that may be made by its manufacturer, is not guaranteed or endorsed by the publisher.
